# Identification of COP9 Signalosome Subunit Genes in *Bactrocera dorsali*s and Functional Analysis of *csn3* in Female Fecundity

**DOI:** 10.3389/fphys.2019.00162

**Published:** 2019-02-26

**Authors:** Jing Zhang, Zhenyu Zhang, Rui Zhang, Wenfei Zhang, Haozhe Li, Tianran Li, Hongyu Zhang, Weiwei Zheng

**Affiliations:** Key Laboratory of Horticultural Plant Biology, Ministry of Education, Hubei Key Laboratory of Insect Resource Application and Sustainable Pest Control, Institute of Urban and Horticultural Entomology, College of Plant Science and Technology, Huazhong Agricultural University, Wuhan, China

**Keywords:** oriental fruit fly, CSN, reproduction, sexual maturation, fecundity, vitellogenin

## Abstract

The COP9 signalosome (CSN) is an evolutionarily conserved multi-subunit complex that plays crucial roles in regulating various biological processes in plants, mammals, and the model insect *Drosophila*. However, it is poorly studied in non-model insects, whereas its role in fecundity remains unclear. In this study, all nine CSN subunits were identified and characterized in *Bactrocera dorsali*s, a major invasive agricultural tephritid pest. Each subunit gene, except for *csn9x1*, encoded a protein containing a PCI/PINT or MPN domain. Phylogenetic analysis revealed that all CSN subunits were individually clustered into a specific branch with their counterparts from other species. All CSN subunit genes were expressed in all detected developmental stages and tissues. Most subunits, except for *csn8* and *csn9x1*, showed the highest expression level in the eggs. Notably, *csn*3 and *csn*5 were significantly enriched in mature female adults. Further analysis of *csn3* revealed that it was enriched in the ovary and that its ovarian expression level gradually increased with the reproductive development process. RNAi-based knockdown of *csn3* in female adults significantly reduced the number of laid eggs. The expression level of *EcRB1* and *USP*, which encode the heterodimer receptors of 20E, and vitellogenin transcripts (*Vg1* and *Vg2*) was suppressed in the fat body of female adults injected with *csn3*dsRNA. Decreased level of Vg1 protein was confirmed by means of Western blots. These data indicate that *csn3* is involved in female reproduction by regulating 20E signaling and Vg synthesis. Overall, our study may facilitate the development of new strategies for controlling *B. dorsali*s since it provides insights into the evolution and expression patterns of all CSN subunit genes as well as the critical roles of *csn3* in female fecundity.

## Introduction

The COP9 signalosome (CSN) is an evolutionarily conserved multi-subunit complex that was originally identified in *Arabidopsis* for the suppression of photomorphogenesis (Wei et al., 1994; Wei and Deng, 2003) and then discovered in a wide range of eukaryotic organisms, including plants, yeast, mammals, and insects (Henke et al., 1999; Oron et al., 2002; Zhou et al., 2003; Tong et al., 2015). Through participating in the ubiquitin-proteasome-mediated protein degradation by interacting with various signaling factors, CSN was considered to have diverse roles in various cellular and developmental processes (Wei and Deng, 2003), including deneddylation (Schwartz and Hochstrasser, 2003), DNA repair (Groisman et al., 2003; Holmberg et al., 2005), cell cycle regulation (Menon et al., 2007; Panattoni et al., 2008), and gene expression (Panattoni et al., 2008). CSN was previously identified as an eight-subunit complex with proteasome-COP9 signalosome-initiation factor 3/proteasome subunits, Int-6, Nip-1, and TRIP-15 (PCI/PINT) or Mov34-Pad-N-Terminal (MPN) domain and named CSN1–CSN8, depending on the gradually decreased molecular weight (Tsuge et al., 2001; Cope et al., 2002; Wei and Deng, 2003; Wei et al., 2008). Recently, CSN9 [or CSN acidic protein (CSNAP)] has been identified in human as the ninth CSN subunit without PCI or MPN domain (Rozen et al., 2015).

CSN subunits are essential for the stability and function of the CSN complex and play multiple roles when combined with other CSN subunits to form homo- or mini-CSN complexes or act independently as free forms (i.e., CSN5) (Dubiel et al., 2015). CSN subunits and their roles have been reported in *Arabidopsis* and mammals. As the largest subunit, CSN1 interacts with other subunits via its large PCI domain and plays a critical role in accumulating CSN subunits for complex stability in *Arabidopsis* (Wang et al., 2002). As one of the most conserved CSN subunits, CSN2 is involved in the proliferation and development of early embryos as well in anti-tumor activity as a tumor suppressor at low levels in mouse (Lykke-Andersen et al., 2003). CSN3 has been confirmed to be essential for the formation and cullin-deneddylating function of the CSN complex as its loss reduces the CSN holo-complex levels, causing developmental defects in *Arabidopsis* and the Smith-Magenis syndrome in human (Peng et al., 2001a; Peth et al., 2007). In addition, CSN3 is necessary for maintaining the cell proliferation of embryonic ectoderm in mouse (Yan et al., 2003). CSN5 (or c-Jun activation domain-binding protein1, JAB1) has been reported as the catalytic center for deneddylation of the Nedd8-cullin in the CSN complex that exists as monomer or a CSN5-containing small complex and plays various roles in HIF1-a stabilization, p27 nuclear export and degradation, E2F1-mediated apoptosis, cell cycle control, and cancer (Wei et al., 2008; Shackleford and Claret, 2010). Different from CSN5, which is a free CSN subunit that occurs under certain circumstances in certain cells, the other CSN subunits, including CSN4 and CSN6, are unlikely released from the CSN holo-complex and probably have limited roles (Dubiel et al., 2015). Although first identified, CSN8 is one of the smallest and least conserved subunits (Wei and Deng, 2003). Deletion of *csn8* in peripheral T cells disrupts the formation of the CSN complex, reduces T cell survival and proliferation *in vivo*, and causes a lack of signal-induced expression of cell cycle-related genes in mouse (Menon et al., 2007). As the only non-PCI or non-MPN subunit, CSN9/CSNAP has been confirmed to incorporate into the CSN complex by binding CSN3, CSN5, and CSN6 and plays critical roles in cellular proliferation and morphology (Rozen et al., 2015). Different CSN variants can interact with different binding partners and substrates, enabling different subunit expression profiles. However, little is known regarding the comprehensive expression profiling of CSN subunits in these species.

In insects, CSN is critical for the development and adult physiology; however, most studies have been conducted in the model species *Drosophila melanogaster* (Lu et al., 2015; Qian et al., 2015; Suisse et al., 2017), in which all CSN subunits have been identified. CSN4 and/or CSN5 were confirmed to play multiple roles in molting, response to DNA damage, and development of the immune system (Oron et al., 2002; Harari-Steinberg et al., 2007). Lack of CSN8 leads to larval lethality as well as the deformation of wings, eyes, and thoracic structures (Oren-Giladi et al., 2008). The reproductive development processes of female insects are very important for species propagation and have significant applications in pest control; however, the involvement of CSN in the reproductive development and fecundity remains unclear.

The oriental fruit fly *Bactrocera dorsalis* is a highly invasive agricultural pest with a wide host range and high fecundity that is currently distributed across most Asian countries and some Pacific islands. Similar to other holometabolous insects, *B. dorsalis* adults emerge from the pupal stage as immature flies and become mature within 10–13 days after nutrition ingestion (Hong and Ding, 2007). Based on our previous transcriptome data of *B. dorsalis*, eight CSN subunits (CSN1–CSN8) were identified, and most of them were found to be differentially expressed between immature and mature female adults (Zheng et al., 2016), indicating that CSN may be involved in the reproductive development of *B. dorsalis*. In this study, we aimed to better understand the spatiotemporal expression profiles of all CSN subunits and their roles in reproduction. To this end, the differential expression of all CSN subunits was validated by quantitative real-time PCR (qRT-PCR), and their mRNA expression profiles in all developmental stages and in different tissues were analyzed. By RNAi-based knockdown, CSN3 was confirmed to play an important role in female fecundity possibly by regulating the expression of vitellogenin. Therefore, this study revealed the crucial roles of CSN in the reproduction of *B. dorsalis* and might facilitate the development of simple and effective pest control strategies.

## Materials and Methods

### Insect Rearing

*Bactrocera dorsalis* samples used in this study were obtained from an established laboratory colony (originated from Guangzhou Province, China and maintained in our lab for at least 13 generations). Insects were cultured in our laboratory at 28°C under a 12-h light: 12-h dark photoperiod. Adults were fed with artificial diets made from yeast extract and sugar (1:3), and the hatched larvae were cultured in bananas (Li et al., 2015).

### Gene Identification and Sequence Analysis

CSN subunit genes were first identified using BLASTN and BLASTX results from our *B. dorsalis* cDNA library (Zheng et al., 2016). Expressed sequence tags (ESTs) showing homology to CSN subunit genes (CSN1- CSN8) were selected for further study. CSN9 was obtained from the GenBank (Sequence ID: XM_011199575.2). The genes were translated, and the domain of the obtained proteins was predicted using the Simple Modular Architecture Research Tool (Letunic and Bork, 2018).

Alignments were performed using DNAMAN v.6.03 (Lynnon Biosoft, San Ramon, CA, United States). Amino acid sequences of CSN genes from *B. dorsalis* were selected for phylogenetic analysis with their counterparts from other insect species (i.e., *D. melanogaster, Ceratitis capitata, Aedes aegypti, Bombyx mori, Apis mellifera*, and *Tribolium castaneum*). *Mus musculus* was also included in the analysis. Phylogenetic trees were constructed using the maximum likelihood method implemented in MEGA 7.0 (Pennsylvania State University, University Park, PA, United States) with the default settings and 1,000 bootstrap replicates.

### Total RNA Isolation and cDNA Synthesis

Total RNA was isolated using TRIzol (Invitrogen, Carlsbad, CA, United States) from immature, mature, and mated female adults to validate the digital gene expression (DGE) data from our previous transcriptome study (Zheng et al., 2016). Flies at 1–2 days after emergence were considered as immature adults, whereas 14-days-old adults without mating were considered virgin adults. To obtain mated flies, 14-days-old virgin female and male adults were placed together shortly before the dark period, and only pairs that mated for at least 90 min were used in further experiments (Zheng et al., 2016). Copulation was terminated naturally, and on the following day (24 h after mating), the flies were chilled briefly. Total RNA was isolated from the whole body of 10 flies.

Total RNA was also extracted from flies at different developmental stages, including eggs, the first instar larvae, the second instar larvae, the third instar larvae, early pupae (1 day after pupation), old pupae (8 days after pupation), immature adults (1 day after eclosion), and virgin adults (15 days after eclosion), as well as from different tissues of 30 mature adults (head, thorax, ovary, testis, gut, and fat body). Additionally, total RNA was isolated from the ovary respectively at 1, 3, 5, 7, 9, 11, 13, 15 days after eclosion.

cDNA synthesis was performed using M-MLV Reverse Transcriptase (First Strand cDNA Synthesis Kit; Takara, Kyoto, Japan). All RNA samples were stored at -80°C and all cDNA samples at -20°C until further use.

### Quantitative Real-Time PCR

Quantitative real-time PCR was performed to detect the expression patterns of CSN subunits with a Bio-Rad real-time thermal cycler using the SYBR Premix ExTaq kit (Bio-Rad, Hercules, CA, United States), according to the manufacturer’s instructions. The PCR program was as follows: 94°C for 3 min followed by 40 cycles at 94°C for 15 s, 60°C for 30 s, and 72°C for 20 s. The melting curve was analyzed to confirm the amplification of a single fragment. The relative gene expression was analyzed by the 2^-ΔΔCt^ method using *rpl32* as an internal reference. The specific primers for qRT-PCR are presented in Supplementary Table S1. Three biological replicates were performed for all PCR experiments.

### dsRNA Synthesis and Microinjection

The target sequence fragment of *csn3* was amplified by nested PCR using primers specific to the T7 RNA polymerase promoter region (*csn3*iF: GGATCCTAATACGACTCACTATAGGCGCATTATGGTAGAGCAA; *csn3*iR: GGATCCTAATACGACTCACTATAGGAATTTCAGACGCCGTAGA). Double-stranded RNA (dsRNA) synthesis was performed using 1 μg of PCR product with the T7 Ribomax Express RNAi System (Promega, Madison, WI, United States), according to the manufacturer’s instructions. The quality and integrity of dsRNA were analyzed by agarose gel electrophoresis, whereas the quantity was determined by a Nanodrop1000 Ultra-micro spectrophotometer (Thermo-Fisher, Waltham, MA, United States).

Needles were prepared with a puller (PC-10; Narishige, Tokyo, Japan) at 60.4°C. Microinjection was performed using an Eppendorf micromanipulation system (FemtoJet 5247; Eppendorf, Hamburg, Germany) as described previously (Yao et al., 2016). Injection conditions were set to a Pi of 300 hpa and a Ti of 0.3 s. Gene knockdown experiments were carried out by injecting 1 μl of *csn3* dsRNA (dscsn3; 2 μg μl^-1^) into the ventral abdomen of female adults at 4 days after eclosion. The flies in the control group were injected with the same amount of *egfp* dsRNA (*egfp*-T7F: GGATCCTAATACGACTCACTATAGGACGTAAACGGCCACAAGTTC; *egfp*-T7R: GGATCCTAATACGACTCACTATAGGAAGTCGTGCTGCTTCATGTG). Total RNA was extracted at 6 days after eclosion to evaluate the knockdown efficiency by qRT-PCR.

### Effects of *csn3* Knockdown on Fecundity

At 10 days after dscsn3 or dsegfp injection, 20 female adults from each group were placed in a new box with the same number of male adults. Next, the number of mating pairs in each group was counted, and the mating rates were calculated. *t-*Tests were performed using Prism 5 (GraphPad, La Jolla, CA, United States) to identify differences in mating rates between dscsn3- and dsegfp-injected female adults. Three biological replicates were performed for each group.

Female adults injected with dscsn3 were placed in a new box (8 cm × 8 cm × 13.5 cm) with male adults for oviposition. Female adults injected with dsegfp were used as a control group. A paper cup (bottom diameter, 3.5 cm; upper diameter, 5 cm; height, 5 cm) was placed in the box for egg laying. For oviposition occurrence, the wall at the middle of the paper cup was randomly perforated nine rows of 10 holes each. Bananas were placed in the paper cups, which were, then, sealed to allow the smell distribution through the holes and induce egg laying. The number of laid eggs in each cup was counted every 2 days for 15 days after copulation. *t*-Tests were performed using Prism 5 to identify differences in fecundity between dscsn3- and dsegfp-injected female adults. Eight biological replicates were performed for each group.

For the hatching rate assay, 20 female adults injected with dscsn3 or dsegfp were placed in new boxes with the same number of male adults. Three biological replicates were performed for each group. Each mating couple was placed in a new box. At 16:00 on the following day, eggs ovulated for 30 min were gently eluted onto a moistened filter paper, picked up with a fine-bristle pen, and placed on a Petri dish covered with wet paper. A total of 40 eggs were placed in each culture dish, and each group was replicated five times. The number of hatched eggs was counted after 48 and 56 h.

### Recombinant Expression and Antibody Preparation of Vg1

The Vg1 cDNA of *B. dorsalis* containing the BamHI and XhoI sites were amplified (ExpF: TACTCAGGATCCGAAGAAGACTACAGTGAATC, ExpR: TACTCACTCGAGGAAAGG ACTCTTTGAATT) and expressed in *E. coli* BL2 (DE3) using the pET32a vector. Rabbit polyclonal antiserum against Vg1 was prepared by affinity chromatography using purified recombinant protein. The specificity of the antiserum was examined by immunoblotting and the antiserum was used in the Western blot experiment.

### Western Blot

Total proteins from fat bodies after RNAi treatment were extracted, quantified, and applied to Western blot analysis based on the previous study (Wang et al., 2017). Protein samples were separated by 12.5% SDS-PAGE and transferred to polyvinylidene difluoride membranes. Rabbit polyclonal antibodies against Vg1 were used at a 1:500 dilution to detect Vg1 proteins followed by the secondary anti-rabbit HRP diluted 1:10000 (Invitrogen). Monoclonal antibodies against β-tubulin (Invitrogen) were used as a loading control.

### Statistical Analysis

One-way analysis of variance was performed in conjunction with Duncan multiple range test for gene expression analysis or Student’s *t*-test for bioassay experiments. Data are presented as means ± standard error.

## Results

### Identification and Analysis of CSN Subunit Genes in *B. dorsalis*

**FIGURE 1:**
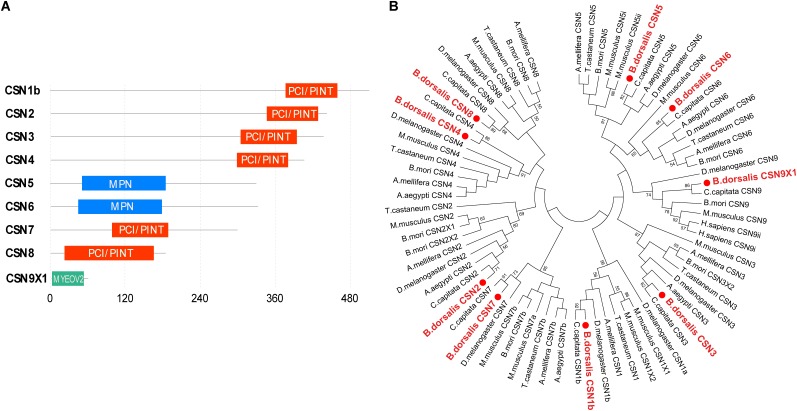
Characterization of *Bactrocera dorsalis* CSN subunit genes. **(A)** Domain structure of CSN proteins with junction amino acid residues. **(B)** Phylogenetic analysis of CSN proteins from *B. dorsalis* (in red solid circles) and their counterparts in *Ceratitis capitata, Drosophila melanogaster, Aedes aegypti, Bombyx mori, Apis mellifera, Tribolium castaneum*, and *Mus musculus.* The maximum likelihood tree was constructed by MEGA 7. Only bootstrap values higher than 50 are shown.

Amino acid sequence analysis revealed that the domain structures of CSN subunits in *B. dorsalis* were consistent with their counterparts in *Drosophila*, human, and *Arabidopsis* (Figure 1A). The C-terminal regions of CSN1b, CSN2, CSN3, CSN4, CSN7, and CSN8 contained a conserved PCI/PINT domain. In CSN 5 and CSN 6, the MPN domain was identified in the N-terminal region, whereas in CSN9, no PCI or MPN domains were found (Figure 1A).

The amino acid sequences of CSN1–CSN9 were subjected to phylogenetic analysis with their homologs in *C. capitata, D. melanogaster, A. aegypti, B. mori, A. mellifera, T. castaneum*, and *M. musculus*. Overall, the homologs of the same CSN protein in different species were clustered in the same branch (Figure 1B). In addition, each CSN subunit of *B. dorsalis* was first clustered in the same group with their counterparts in *C. capitata* (amino acid identity of 92% for CSN1, 99% for CSN2, 91% for CSN3, 99% for CSN4, 95% for CSN5, 91% for CSN6, 84% for CSN7, 78% for CSN8, and 100% for CSN9; Figure 1B and Supplementary Figure S1 and Supplementary Table S2), which was consistent with the nearest evolutionary distance of these two species in Tephritidae. The amino acid sequence alignment of each CSN subunit among *B. dorsalis* and other dipteran species were also constructed together in the same or nearer branch, revealing the conserved amino acid sequences of CSN proteins in Diptera (Figure 1B).

### Enhanced Expression of CSN Subunit Genes in the Reproductive Stage of Female Adults

**FIGURE 2:**
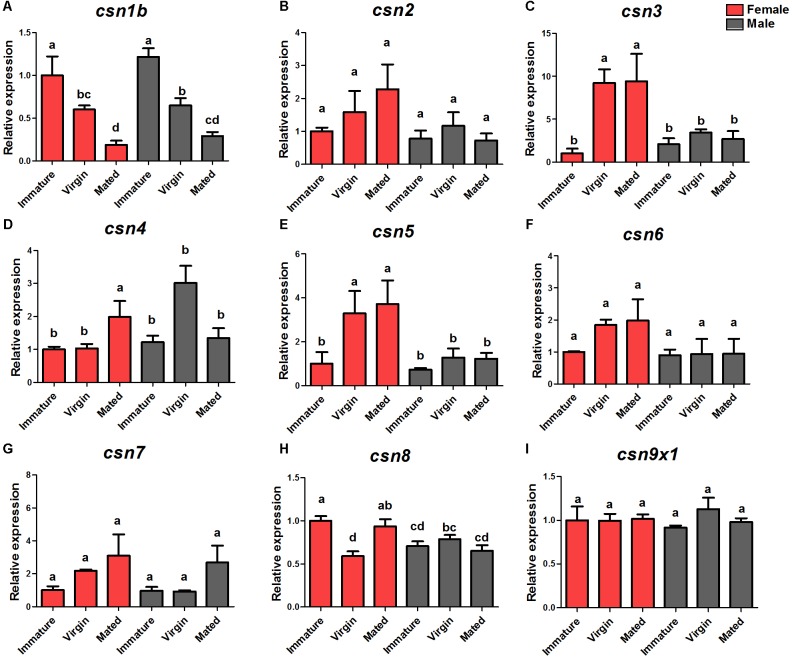
Expression profiles of CSN subunit genes at female/male adult stages of *B. dorsalis* **(A–I)**. Total RNA was extracted from immature adults (newly emerged within 24 h), virgin adults (14-days-old adults before mating), and mated adults (14-days-old adults post-mating). *rpl32* was used as an internal reference. Three biological replicates were performed for each group. Different letters indicate significant differences at *P* < 0.05.

The DEG analysis of our previously established cDNA library revealed eight CSN genes, of which six (*csn2, csn3, csn4, csn5, csn6, csn7*) were up-regulated in virgin female adults compared with their expression in immature female adults (Zheng et al., 2016). To verify the enhanced expression of CSN subunit genes during the sexual maturation of female adults, detailed expression profiles of the adult stage of both genders were detected by qRT-PCR. The results were consisted with transcriptome data and confirmed that *csn3* and *csn5* were significantly up-regulated in virgin and mated female adults (Figure 2C,E). Similarly, the expression levels of *csn2, csn4, csn6*, and *csn7* reached a peak in virgin and mated female adults without significant differences (Figure 2B,D,F,G), which could be attributed to the low fold (<2) of DEG data. In male flies, the expression level of all subunits was steady in all three studied stages (Figure 2). Additionally, *csn8* and *csn9x1* showed a steady expression level along the reproductive development stages of both genders (Figure 2H,I).

### Specific Expression Profiles of Nine CSN Subunit Genes During Development

**FIGURE 3:**
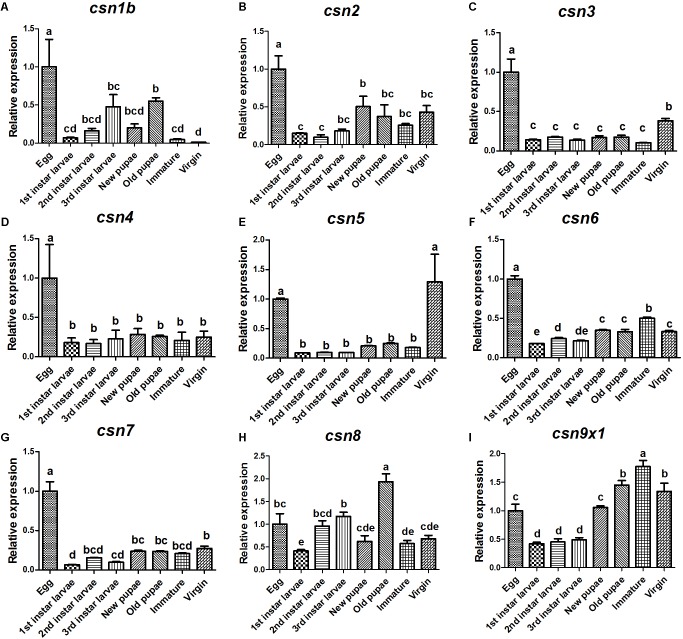
Expression profiles of CSN subunit genes at different developmental stages of *B. dorsalis* **(A–I)**. Total RNA was extracted from egg, 1st instar larvae, 2nd instar larvae, 3rd instar larvae, new pupa (1 day after pupation), old pupa (8 days after pupation), immature adults (1 day after eclosion), and virgin adults (15 days after eclosion). *rpl32* was used as an internal reference. Three biological replicates were performed for each group. Different letters indicate significant differences at *P* < 0.05.

The expression profiles of nine CSN subunit genes were further studied in the stages of egg, 1st, 2nd, and 3rd larvae, new pupae, old pupae, immature female adults, and mature virgin female adults to detect any patterns related to development. The results showed that these subunits shared a similar expression pattern and also had unique profiles. All CSN subunit genes were expressed in all studied stages and reached a peak in the eggs, except for *csn8* and *csn9x1* (Figure 3). Notably, *csn*3 and *csn*5 were significantly enriched in mature female adults (Figure 3C,E). *csn2, csn4, csn6*, and *csn7* were stably expressed from larvae to adult stage (Figure 3B,D,F,G).

### *csn3* Was Highly Expressed in the Ovary of Female Adults During Sexual Maturation

**FIGURE 4:**
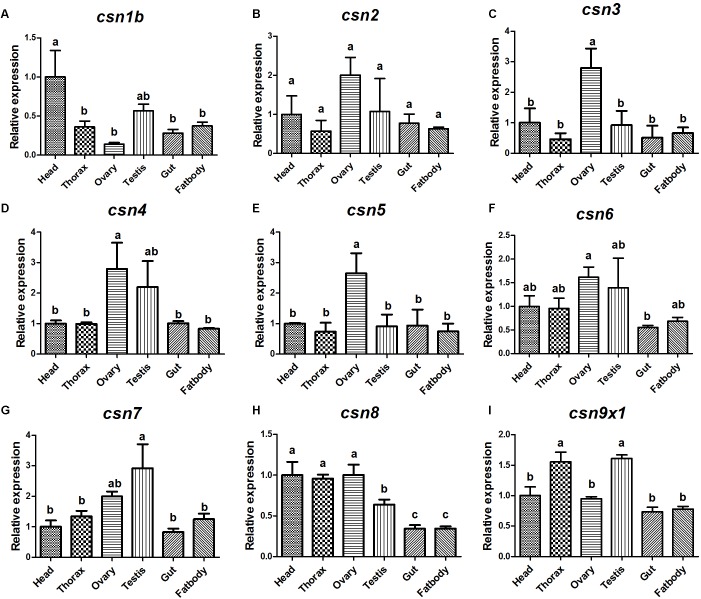
Expression profile of CSN subunit genes in different tissues of mature adults in *B. dorsalis* **(A–I)**. Total RNA was isolated from head, thorax, ovary, testis, gut, and fat body. Different letters indicate significant differences at *P* < 0.05.

The expression pattern of CSN subunit genes in different tissues were further analyzed by qRT-PCR. The results showed that all CSN subunit genes were expressed in all studied tissues (head, thorax, ovary, testis, gut, and fat body), and most of them were abundantly expressed in the reproductive tissues, ovary and (or) testis (Figure 4). Notably, *csn3* and *csn5* showed the significantly higher expression level in the ovary than in any other tissues (Figure 4C,E). The expression level of *csn4* and *csn6* was higher in the reproductive tissues than in other tissues (Figure 4D,F). Besides, *csn7* was abundantly expressed in the testis (Figure 4G).

**FIGURE 5:**
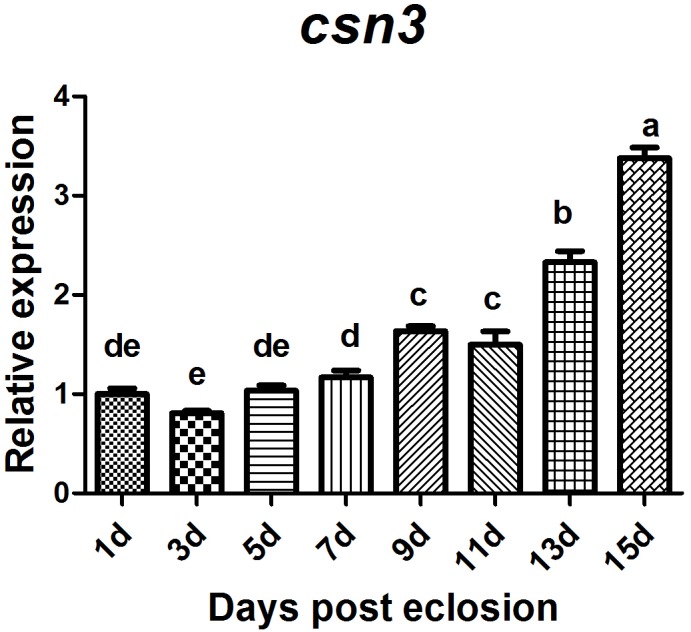
Relative expression of *csn3* in the ovary during sexual maturation in *B. dorsalis*. Total RNA was extracted from the ovary at 1, 3, 5, 7, 9, 11, 13, and 15 days after elosion. Different letters indicate significant differences at *P* < 0.05.

Of *csn3* and *csn5* that were found to be highly expressed in the ovary of mature female adults, the former was selected for further study to investigate the role of CSN complex and the subunits in reproduction because it is still unclear whether *csn3* is involved in animal reproduction. The sexual maturation process lasts 14 days under our rearing conditions. To compare the temporal profile of *csn3* in the ovary during sexual maturation, *csn3* was analyzed at 1, 3, 5, 7, 9, 11, 13, and 15 days after eclosion. The results showed that the expression level gradually increased with the reproductive development and was dramatically upregulated at 13 and 15 days after eclosion (Figure 5).

### RNAi-Based Knockdown of *csn3* Reduced the Fecundity of Female Adults

**FIGURE 6:**
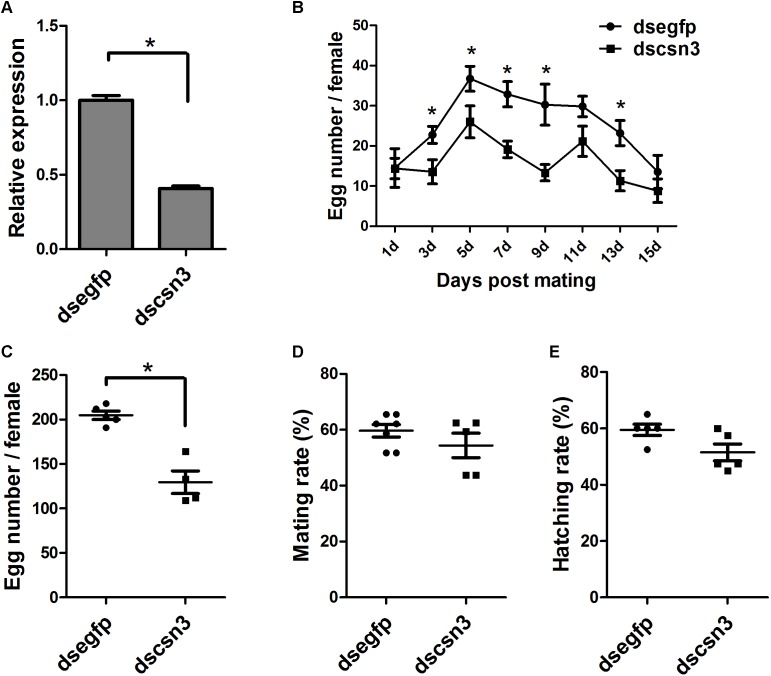
Effects of RNAi-based knockdown of *csn3* on the fecundity of *B. dorsalis*. **(A)** *csn3* transcription levels in the ovary of dscsn3-injected and dsegfp-injected female adults. **(B)** Number of laid eggs during oviposition. **(C)** Total number of laid eggs, **(D)** mating rate, and **(E)** egg hatching rate of dscsn3-injected and dsegfp-injected female adults. ^∗^ Indicates significant differences between dscsn3-injected and dsegfp-injected female adults at *P* < 0.05.

The enhanced expression of *csn3* in the ovary of female adults during sexual maturation revealed its important role in reproduction. Therefore, RNAi-based knockdown experiments were conducted to reveal the function of *csn3* in reproduction. As shown in Figure 6A, the *csn3* mRNA levels were 60% lower in the ovary of dscsn3-injected female adults than those in the ovary of dsegfp-injected female adults (*P* < 0.05).

The knockdown of *csn3* significantly reduced egg laying during the oviposition period (3, 5, 7, 9, and 13 days after mating), resulting in a decreased number of laid eggs by 43% (Figure 6B,C). The ovarian development of dscsn3-injected adults was examined at 3, 6, and 9 days after injection, but no detectable alterations were identified in the ovary length or width (data not shown). Additionally, the copulation and progeny viability did not change in dscsn3-injected female adults (Figure 6D,E).

### *csn3* RNAi Depletions Resulted in the Decreased Level of Vg Protein in the Fat Body of Adult Females

**FIGURE 7:**
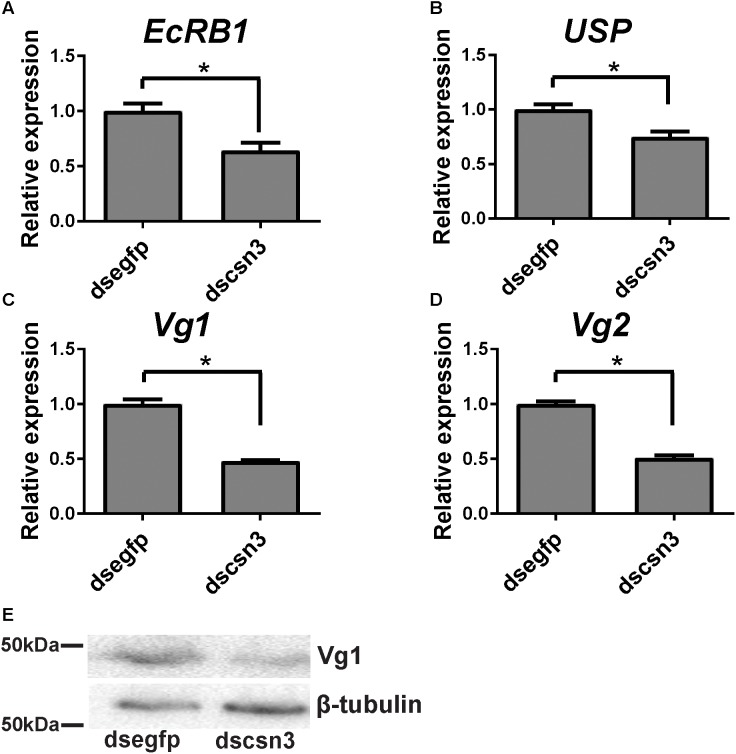
Knockdown of *csn3* resulted in decreased gene transcription in 20E signaling and Vg protein in the female fat body of *B. dorsalis*. Relative expression levels of **(A)** *EcRB1*, **(B)** *USP*, **(C)** *Vg1*, and **(D)** *Vg2* at 2 days after injection. **(E)** Knockdown of *csn3* suppressed the abundance of Vg proteins. Rabbit polyclonal antibodies against β-tubulin were used as a loading control. Shown are Western blot analyses using antibodies against Vg1 or β-tubulin (as control). ^∗^ Indicates significant difference between dscsn3-injected and dsegfp-injected female adults at *P* < 0.05.

During vitellogenesis of dipterans, Vg [the major yolk protein precursors (YPPs)] is synthesized in and secreted from the fat body regulated by 20E signaling pathways and is subsequently accumulated in developing oocytes (Roy et al., 2018). To better understand the underlying mechanism of *csn3* knockdown in fecundity, we further examined the expression levels of *EcRB1* and *USP*, which encode the heterodimer receptors of 20E, in the fat bodies of dscsn3/dsegfp-injected female adults. Also, the expression levels of two Vg genes (*Vg1* and *Vg2*) and the Vg1 protein were detected. The results showed that depletion of *csn3* significantly suppressed the abundance of *EcRB1, USP* as well as Vg transcripts (Figure 7A–D). Depletion of Vg proteins was confirmed by means of Western blots (Figure 7E). This suggests that *csn3* might control fecundity by regulating 20E action and then vitellogenin synthesis, which is essential for the successful female reproduction in *B. dorsalis*.

## Discussion

The expression profile analysis of all subunits in the *B. dorsalis* COP9 signalosome showed that CSN is abundantly expressed in mature female adults. Furthermore, our results revealed that *csn3* was essential for female fecundity. To our knowledge, this is the first report of comprehensively profiling the spatiotemporal expression of CSN subunit genes and studying their effect on fecundity.

CSN subunit genes were named *csn1–csn8* in most plants, animals, and fungi, according to peptide band positions in gel filtration columns depending on the gradually decreased amount of amino acid residues (Wei and Deng, 2003). In multiple species, including vertebrate animals, flies, plants, and fission yeast, the c-terminal regions of CSN1, CSN2, CSN3, CSN4, CSN7, and CSN8 contain a conserved PCI/PINT domain, which is considered necessary for the interactions between CSN subunits and therefore, may function as a scaffold for the CSN complex assembly or other binding partners (Tsuge et al., 2001; Wei and Deng, 2003). For CSN 5 and CSN 6, the MPN domain was identified in the N-terminal region. The MPN domain of CNS5 includes a metalloprotease motif, known as Jab1/MPN domain metalloenzyme (JAMM) or MPNC motif, which facilitates CSN5 to act as a catalytic center for the deneddylation of Nedd8-cullin in the CSN complex (Cope et al., 2002; Wei et al., 2008). The molecular weight and domains of CSN subunits in *B. dorsalis* were similar with those in other species, indicating that CSN and its subunits might be evolutionarily conserved. Additionally, our phylogenetic analysis showed the orthologous relationships of CSN subunits in *B. dorsalis* with those in other model insect species as well as their high similarity in terms of amino acid sequences, revealing conserved peptide structures with orthologous functions in insects.

Further comprehensive investigation of the spatiotemporal expression patterns of all CSN subunits in *B. dorsalis* showed that they were expressed at all developmental stages, indicating that they might be involved in diverse physiological processes at all developmental stages by regulating protein stability through deneddylation or deubiquitination, protein phosphorylation, or subcellular distribution, similar as in mammals and *Drosophila* (Freilich et al., 1999; Kato and Yoneda-Kato, 2009). To our knowledge, this is the first report that presents the expression profiles of all nine CSN subunits throughout the lifespan of both female and male individuals in animals. All CSN subunits were abundantly expressed in eggs, implying their critical role in the embryonic development of *B. dorsalis*, similar as in mammals and *Drosophila* (Lykke-Andersen et al., 2003; Yan et al., 2003; Tomoda et al., 2004). In addition, most subunits exhibited the similar expression pattern during development in different tissues. It implies that these subunits should perform a typical CSN complex with the core subunit CSN5 as the catalytic center for multifunction in regulating various biological processes as that in other animals (Wei and Deng, 2003; Wei et al., 2008). However, some subunits showed varied expression patterns in different developmental stages (i.e., *csn1* and *csn8*), probably because they might function as a complex and also play diverse roles either independently or as a mini-complex via interacting with different binding partners and substrates.

During sexual maturation, dramatic changes take place at the transcriptional level in insects (Gomulski et al., 2012; Zheng et al., 2016). In our previous study, many genes were found to be differentially expressed between immature and mature female adults (Zheng et al., 2016). In the present study, we found that *csn3* and *csn*5 were significantly upregulated in mature female adults compared with that in immature adults and that *csn3* and *csn5* was enriched in the ovary, suggesting that they might play important roles in the reproductive development of female adults. During this progress, CSN5 might perform as the catalytic center of CSN holocomplex that functions in ubiquitin-proteasome pathway through deneddylation, or stabilize/unstablize its binding partners in form of monomeric CSN5 or CSN5-containing mini-complex (Wei et al., 2008). In terms of CSN3, it has been confirmed to be critical for formation of the CSN complex with the function in cullin-deneddylation, and its reduction results in the CSN complex destruction and cell death (Dubiel et al., 2015). So we predicted that the CSN3 might act as the critical regulator of female reproduction through stabilizing the CSN5-containing CSN holocomplex or mini-complex. Similar function of CSN in fecundity has also been reported in *Arabidopsis* and *Drosophila*. In *Arabidopsis*, CSN subunits are highly enriched in floral tissues during flower development, whereas CSN1 or CSN6 partially deficient mutants exhibit an aberrant development of floral organs and low fertility (Peng et al., 2001b; Wang et al., 2003). In *Drosophila*, several CSN subunits, including CSN5, are critical for the germline stem cell differentiation in the ovary and testis (Lu et al., 2015; Qian et al., 2015). This is also consistent with our results that most CSN subunit genes were enriched in the reproductive tissues of *B. dorsalis*.

In the present study, *csn3* was confirmed to be required for female fecundity and consequently, for a successful reproduction in *B. dorsalis*. In *Drosophila, csn4* and *csn5* are considered essential for oogenesis since mutants have defective oocytes and embryos (Oron et al., 2002; Doronkin et al., 2003). Here, we confirmed the important role of *csn3* in the female fecundity of *B. dorsalis*; however, further research is needed to detect whether the decreased number of laid eggs is caused by abnormal oogenesis. Additionally, we found that *csn3* knockdown suppressed the expression level of *EcRB1, USP, Vg1*, and *Vg2* transcripts as well as Vg1 proteins in the fat body. 20E is the principal hormone regulating female reproduction in dipterans. During sexual maturation, the synthesis of vitellogenin in fat body and its uptake by maturing oocytes are essential biological events for a successful female reproduction (Roy et al., 2018). The transcription of *Vg* is controlled by 20E signaling in the fat body of *B. dorsalis* and some other insects (Tufail and Takeda, 2008; Chen et al., 2012; Roy et al., 2018). The Vg 5′ regulatory region contains several EcREs (20E response element, EcRE), providing evidence of direct control of this gene by EcR–USP (Kokoza et al., 2001). Thus, *csn3* might regulate the female oviposition through the 20E signaling that targets the Vg synthesis. In fact, several CSN subunits, including CSN2/Alien, CSN4, and CSN5, have been demonstrated to interact with hormone pathways (i.e., 20E signaling pathway in *Drosophila*; Dressel et al., 1999; Huang et al., 2014). CSN4 and CSN5 regulate the 20E signaling pathway through its association with ecdysone receptors that cooperate with the deneddylation machinery and temporally shutdown the downstream target gene expression (i.e., *br* that encodes the BR-Z1 transcription factor activates neural competence; Huang et al., 2014). Our results suggested that *csn3* might play critical roles in controlling the female reproduction through the 20E signaling pathway; however, further research is needed to better explore the underlying mechanism of CSN subunits in the regulation of female fecundity.

In summary, this study significantly advances the characterization of CSN subunits and the roles in reproduction in the oriental fruit fly, a major invasive agricultural tephritid pest. We identified and characterized all CSN subunits in *B. dorsalis*, revealed the phylogenetic evolution and comprehensive expression profiles of CSN subunits. The spatiotemporal expression patterns showed that *csn3* was highly expressed in the ovary of female adults during sexual maturation. RNAi-based knockdown of *csn3* indicated that it might play an essential role in female fecundity. Furthermore, our results shed light on the diverse physiological functions of CSN that might facilitate the development of effective and eco-friendly pest control strategies.

## Data Availability

The datasets generated for this study can be found in NCBI, TSA No. GEEA00000000.

## Author Contributions

WWZ conceived and designed the study. JZ and RZ conducted the RNA extraction and performed qRT-PCR. ZYZ performed the sequence analysis. JZ, ZYZ, TRL, and HZL performed RNAi experiments. WFZ conducted the recombinant expression and Western blot analysis. JZ, ZYZ, and WWZ prepared the initial draft of the manuscript. HYZ approved the final version.

## Conflict of Interest Statement

The authors declare that the research was conducted in the absence of any commercial or financial relationships that could be construed as a potential conflict of interest.

## Supplementary Material

FIGURE S1 Multiple alignments of CSN1-CSN9 amino acid sequences with those of the corresponding genes obtained from other insects. **(A)** CSN1: *Bactrocera cucurbitae* (XP_011188906.1), *Ceratitis capitata* (XP_004518702.1), *Musca domestica* (XP_005177221.1), *D. melanogaster* (NP_524152.2), *A. aegypti* (XP_021699402.1). **(B)** CSN2: *B. cucurbitae* (XP_011189326.1), *C. capitata* (XP_004533981.1), *M. domestica* (XP_005177843.1), *D. melanogaster* (NP_523517.1), *A. aegypti* (XP_001651398.1). **(C)** CSN3: *Bactrocera latifrons* (XP_018783863.1), *Bactrocera oleae* (XP_014095548.1), *B. cucurbitae* (XP_011181361.1), *C. capitata* (XP_004520211.1), *M. domestica* (XP_005179912.1), *D. melanogaster* (AAD28606.1), *A. aegypti* (XP_001661171.1). **(D)** CSN4: *B. oleae* (XP_014092265.1), *B. cucurbitae* (XP_011190651.1), *C. capitata* (XP_004522580.1), *M. domestica* (XP_005178420.1), *D. melanogaster* (NP_477444.1), *A. aegypti* (XP_001654142.1). **(E)** CSN5: *B. cucurbitae* (XP_011182476.1), *B. latifrons* (XP_018790523.1), *B. oleae* (XP_014089194.1), *C. capitata* (XP_004524029.1), *M. domestica* (XP_011292660.1), *A. aegypti* (XP_001649479.2), *D. melanogaster* (NP_477442.1). **(F)** CSN6: *B. latifrons* (XP_018804613.1), *B. oleae* (XP_014088126.1), *C. capitata* (XP_004523378.1), *D. melanogaster* (NP_524451.1), *M. domestica* (XP_005186161.1), *A. aegypti* (XP_001648346.1). **(G)** CSN7: *B. latifrons* (XP_018792029.1), *B. oleae* (XP_014099043.1), *B. cucurbitae* (XP_011184385.1), *C. capitata* (XP_004534617.1), *D. melanogaster* (AAS93704.1), *A. aegypti* (ABF18428.1). **(H)** CSN8: *B. cucurbitae* (XP_011189353.1), *C. capitata* (XP_004521454.1), *M. domestica* (XP_005190089.1), *D. melanogaster* (NP_723378.2), *A. aegypti* (XP_001652939.1). **(I)** CSN9: *B. cucurbitae* (XP_011184186.1), *C. capitata* (XP_012159048.1), *M. domestica* (XP_005176984.1). , identity = 100%; , identity ≥ 75%; , identity ≥ 50%.

TABLE S1 Primers used for gene expression profile analysis of CSN subunit genes by qRT-PCR.

TABLE S2 CSN subunit genes with best-hit matches to dipteran gene counterparts.
