# Supplementary figures and images for: Identification of COP9 Signalosome Subunit Genes in Bactrocera dorsalis and Functional Analysis of csn3 in Female Fecundity
Source: Front Physiol. 2019 Feb 26;10:162. doi: 10.3389/fphys.2019.00162 (PMC6399477; doi:10.3389/fphys.2019.00162)

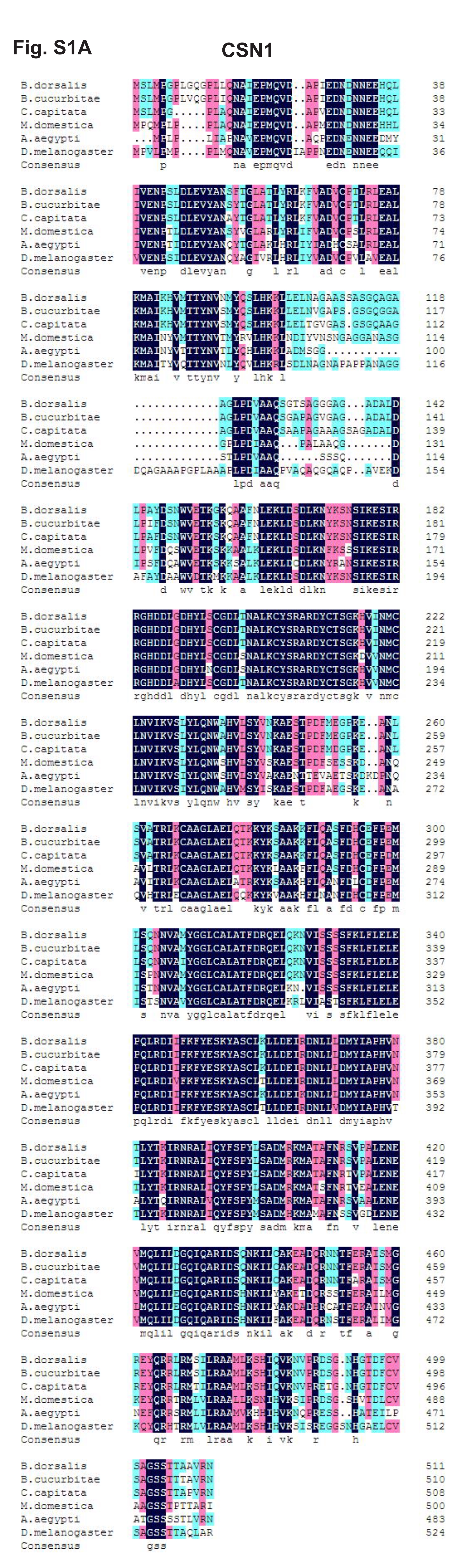

Supplement: FIGURE S1 — Multiple alignments of CSN1-CSN9 amino acid sequences with those of the corresponding genes obtained from other insects. (A) CSN1: Bactrocera cucurbitae (XP_011188906.1), Ceratitis capitata (XP_004518702.1), Musca domestica (XP_005177221.1), D. melanogaster (NP_524152.2), A. aegypti (XP_021699402.1). (B) CSN2: B. cucurbitae (XP_011189326.1), C. capitata (XP_004533981.1), M. domestica (XP_005177843.1), D. melanogaster (NP_523517.1), A. aegypti (XP_001651398.1). (C) CSN3: Bactrocera latifrons (XP_018783863.1), Bactrocera oleae (XP_014095548.1), B. cucurbitae (XP_011181361.1), C. capitata (XP_004520211.1), M. domestica (XP_005179912.1), D. melanogaster (AAD28606.1), A. aegypti (XP_001661171.1). (D) CSN4: B. oleae (XP_014092265.1), B. cucurbitae (XP_011190651.1), C. capitata (XP_004522580.1), M. domestica (XP_005178420.1), D. melanogaster (NP_477444.1), A. aegypti (XP_001654142.1). (E) CSN5: B. cucurbitae (XP_011182476.1), B. latifrons (XP_018790523.1), B. oleae (XP_014089194.1), C. capitata (XP_004524029.1), M. domestica (XP_011292660.1), A. aegypti (XP_001649479.2), D. melanogaster (NP_477442.1). (F) CSN6: B. latifrons (XP_018804613.1), B. oleae (XP_014088126.1), C. capitata (XP_004523378.1), D. melanogaster (NP_524451.1), M. domestica (XP_005186161.1), A. aegypti (XP_001648346.1). (G) CSN7: B. latifrons (XP_018792029.1), B. oleae (XP_014099043.1), B. cucurbitae (XP_011184385.1), C. capitata (XP_004534617.1), D. melanogaster (AAS93704.1), A. aegypti (ABF18428.1). (H) CSN8: B. cucurbitae (XP_011189353.1), C. capitata (XP_004521454.1), M. domestica (XP_005190089.1), D. melanogaster (NP_723378.2), A. aegypti (XP_001652939.1). (I) CSN9: B. cucurbitae (XP_011184186.1), C. capitata (XP_012159048.1), M. domestica (XP_005176984.1). , identity = 100%; , identity ≥ 75%; , identity ≥ 50%. [file Image_1.JPEG]

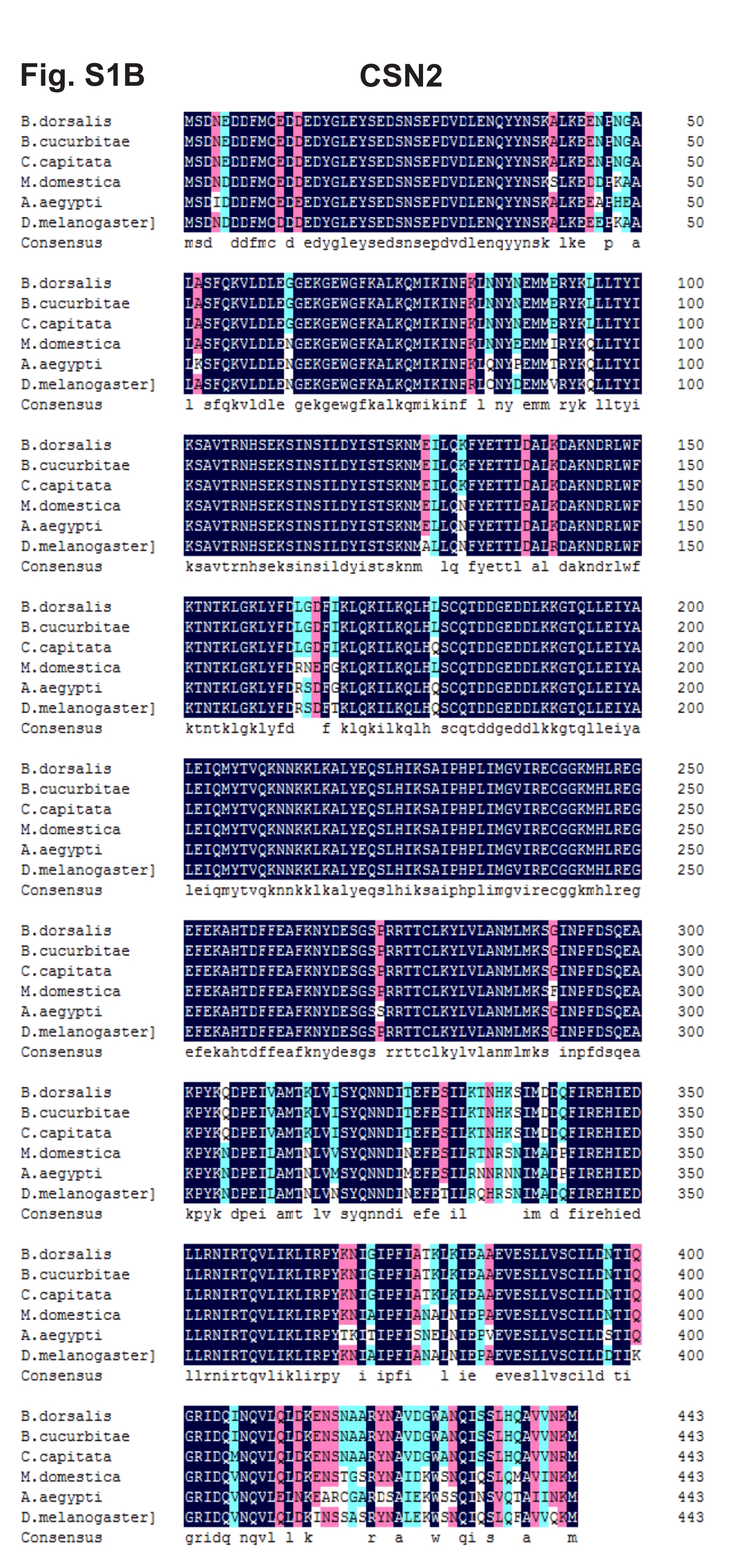

Supplement: Supplementary file 2 [file Image_2.JPEG]

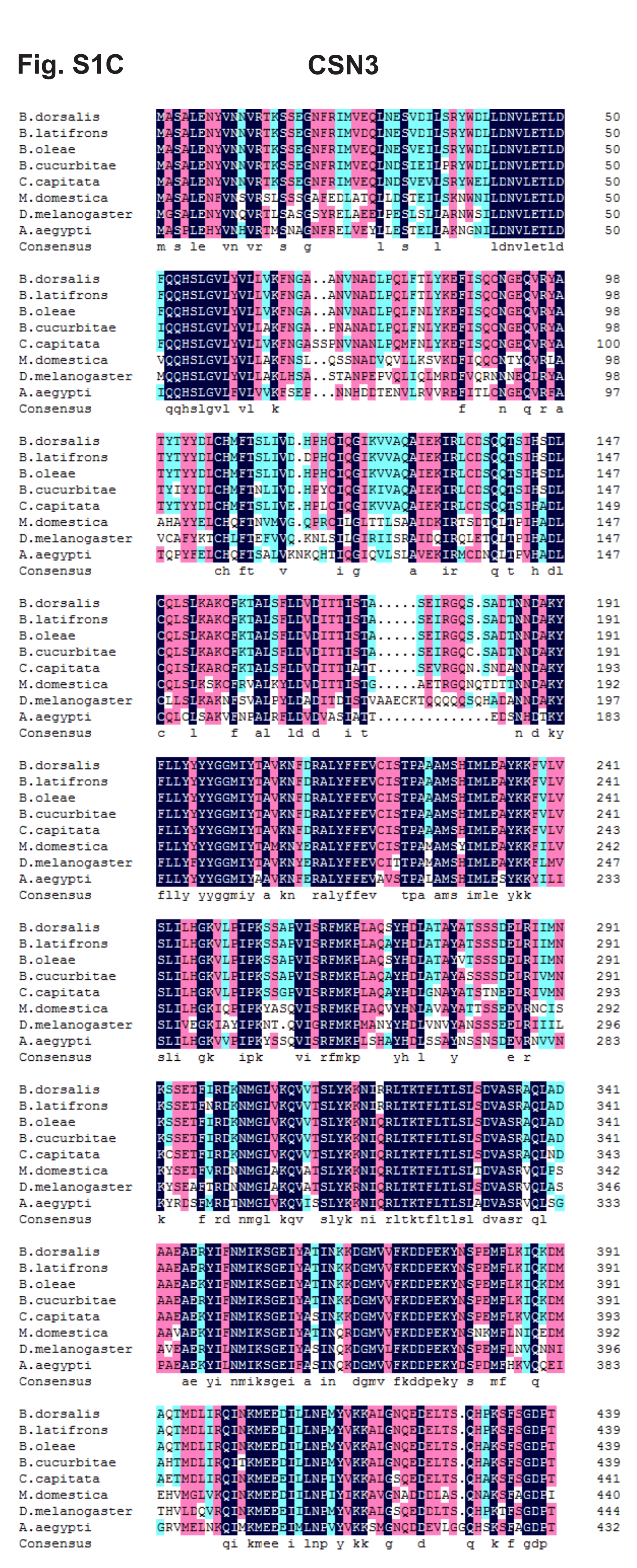

Supplement: Supplementary file 3 [file Image_3.JPEG]

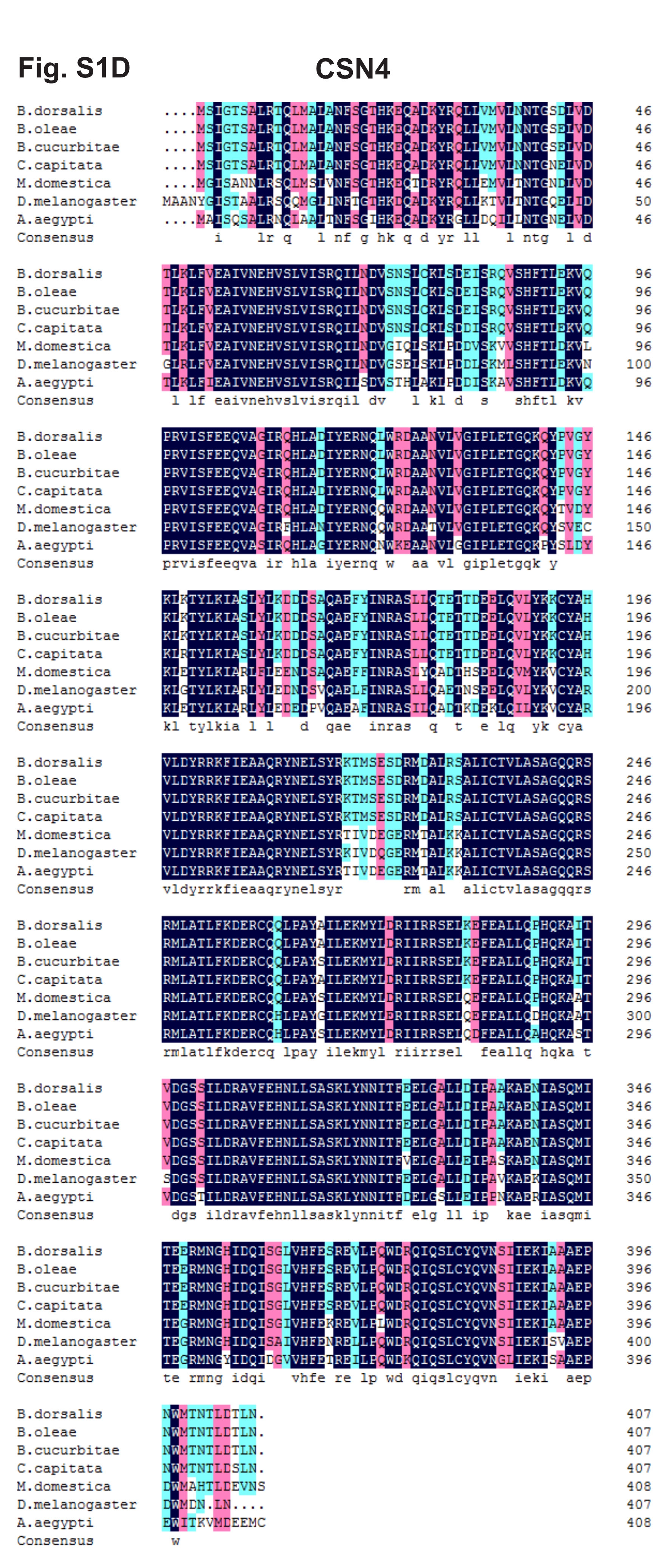

Supplement: Supplementary file 4 [file Image_4.JPEG]

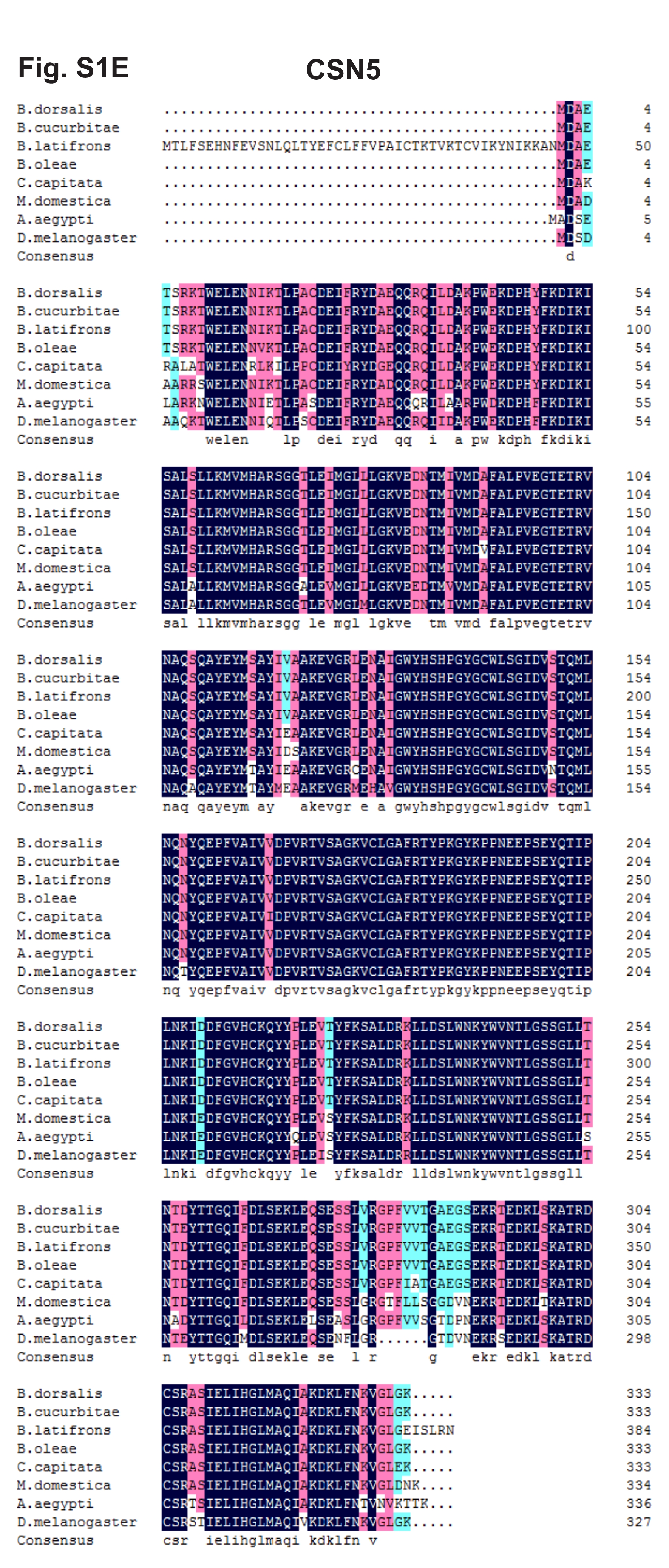

Supplement: Supplementary file 5 [file Image_5.JPEG]

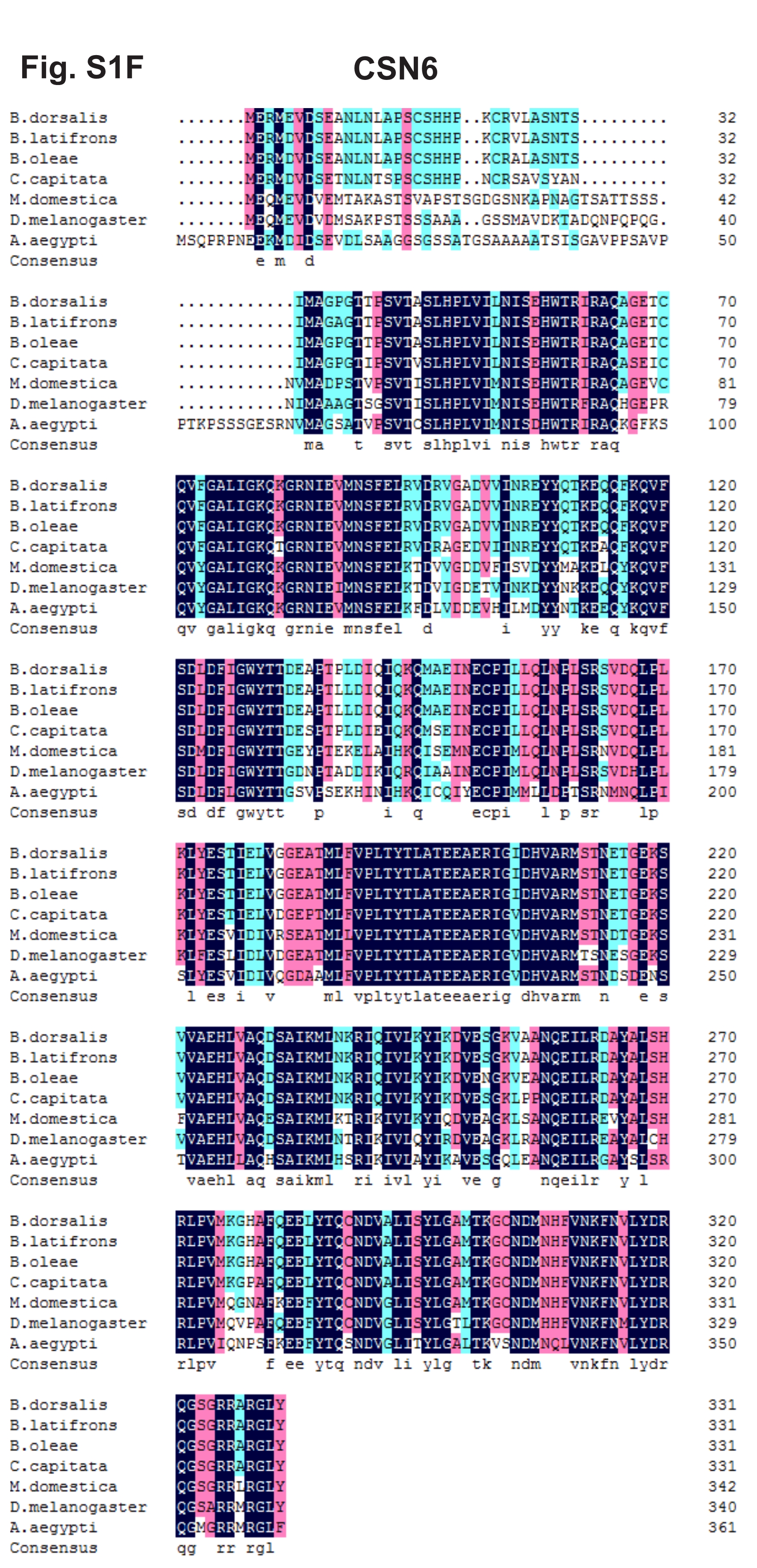

Supplement: Supplementary file 6 [file Image_6.JPEG]

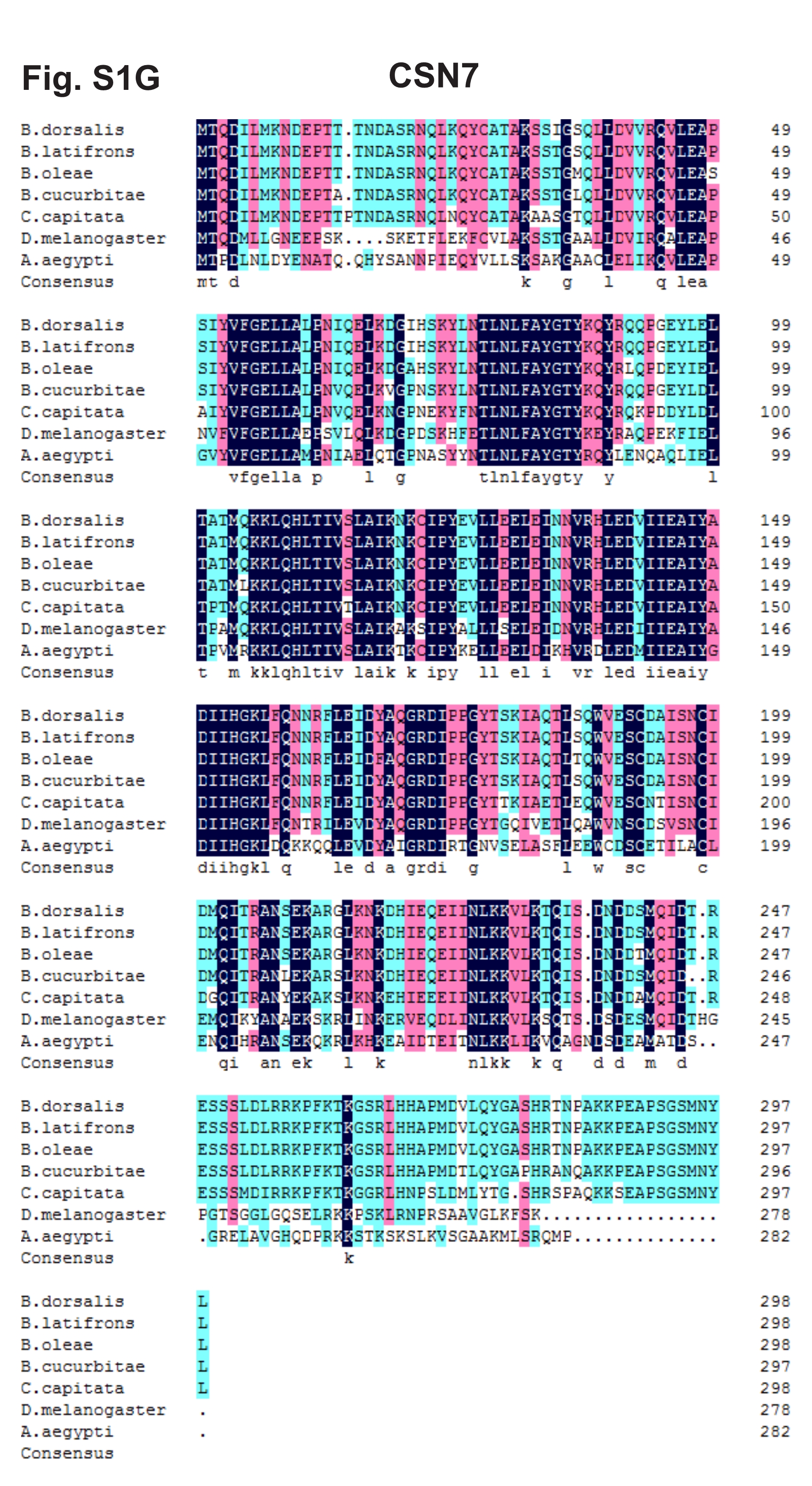

Supplement: Supplementary file 7 [file Image_7.JPEG]

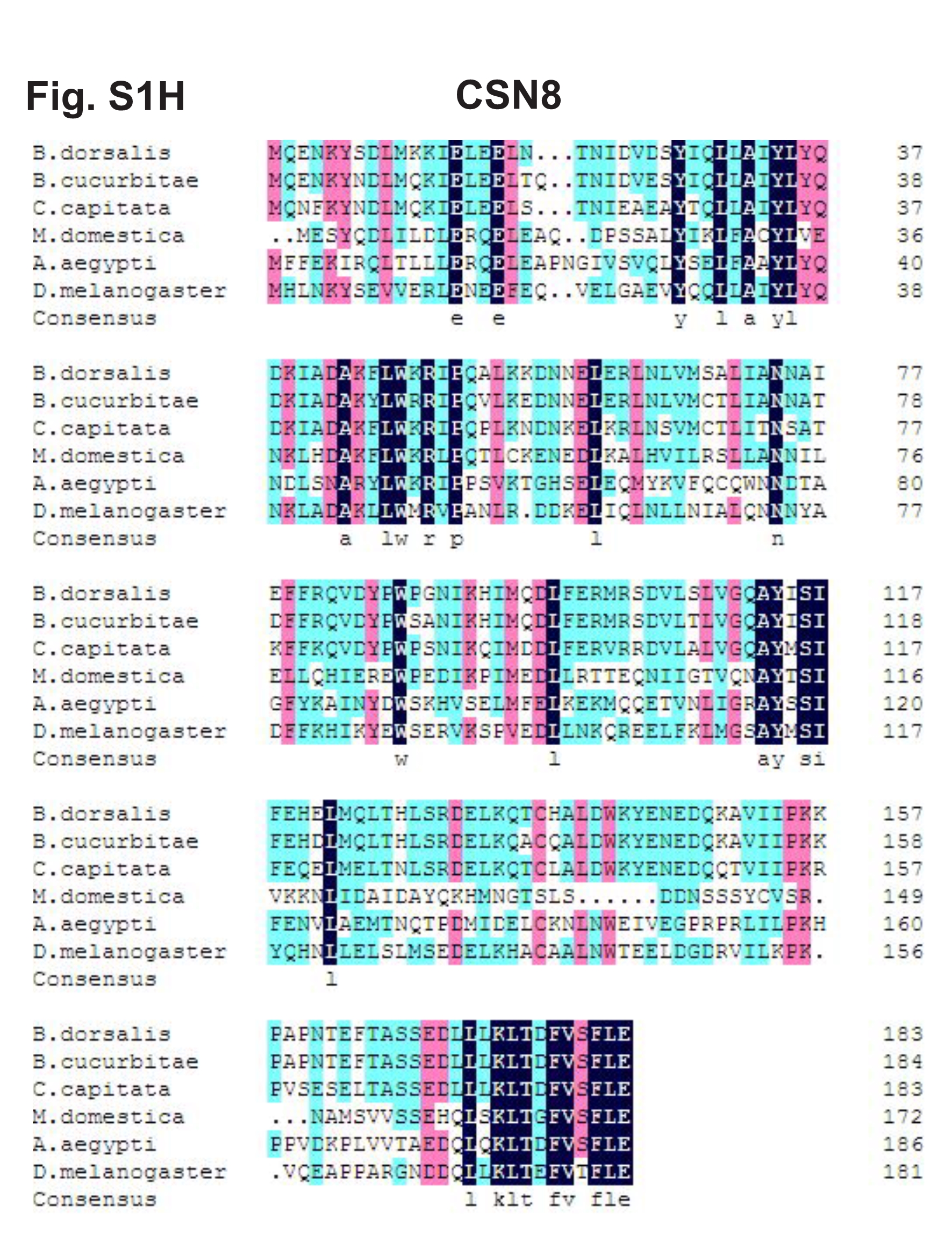

Supplement: Supplementary file 8 [file Image_8.JPEG]

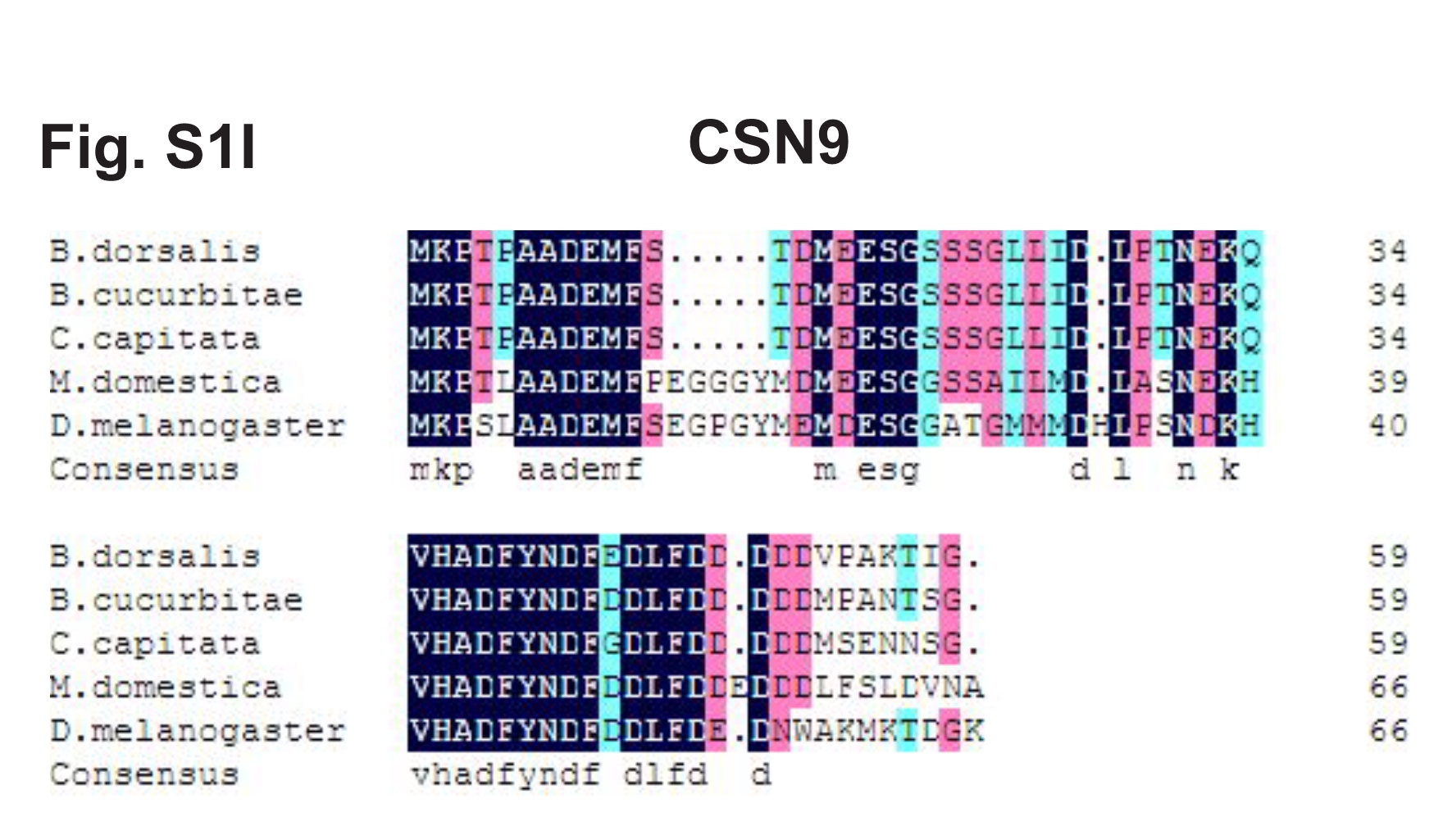

Supplement: Supplementary file 9 [file Image_9.JPEG]
