# Supplementary material for: Identification of COP9 Signalosome Subunit Genes in Bactrocera dorsalis and Functional Analysis of csn3 in Female Fecundity
Source: Front Physiol. 2019 Feb 26;10:162. doi: 10.3389/fphys.2019.00162 (PMC6399477; doi:10.3389/fphys.2019.00162)
Supplement: TABLE S1 — Primers used for gene expression profile analysis of CSN subunit genes by qRT-PCR. [file Table_1.DOCX]

**Table S1. Primers used for gene expression profile analysis of CSN subunit genes by qRT-PCR.**

| **Primer name** | **(5′→3′) nucleotide sequence** |
| --- | --- |
| *csn1b*RTF | GGACAGGGACCTTTGCTACA |
| *csn1b*RTR | AAACAACGGACAAAGGGTGG |
| *csn2*RTF | TGGCATACCATTCATAGCG |
| *csn2*RTR | TTGTTGACCACAGCCTGAT |
| *csn3*RTF | TTCGCTGGGAGTGCTGTA |
| *csn3*RTR | GTTCGCCGTTGCATTGAC |
| *csn4*RTF | GACCGAGACAACCGATGAAG |
| *csn4*RTR | GATGCTAACACCGTGCAAAT |
| *csn5*RTF | AGCCACCGAATGAAGAACCT |
| *csn5*RTR | GTAAGCCCGAACTACCCAGT |
| *csn6*RTF | ACCATCTGTGACCGCCTCT |
| *csn6*RTR | CACCCACTCTGTCCACTCTTAA |
| *csn7*RTF | TGCTGATATTATACACGGCAAAC |
| *csn7*RTR | CTCCACCCACTGCGATAGG |
| *csn8*RTF | GCCATGCATTAGACTGGAAA |
| *csn8*RTR | TCTTCCGAACTTGCTGTGAA |
| *csn9x1*RTF  *csn9x1*RTR | ATGAAGCCAACACCGGCT  CTGGCACATCATCGTCATC |
| *rpl32*RTF | CCCGTCATATGCTGCCAACT |
| *rpl32*RTR | GCGCGCTCAACAATTTCCTT |
