# Supplementary material for: Identification of COP9 Signalosome Subunit Genes in Bactrocera dorsalis and Functional Analysis of csn3 in Female Fecundity
Source: Front Physiol. 2019 Feb 26;10:162. doi: 10.3389/fphys.2019.00162 (PMC6399477; doi:10.3389/fphys.2019.00162)
Supplement: TABLE S2 — CSN subunit genes with best-hit matches to dipteran gene counterparts. [file Table_2.DOCX]

**Table S2. CSN subunit genes with best-hit matches to dipteran gene counterparts.**

| **Gene name** | **ORF Length**  **(aa)** | **Species** | **Subject ID** | **E-value** | **Identity (%)** |
| --- | --- | --- | --- | --- | --- |
| *csn1b* | 511 | *C.capitata* | XP_004518702.1 | 0 | 92 |
|  |  | *D. melanogaster* | AAD28605.1 | 0 | 73 |
| *csn2* | 444 | *C.capitata* | [XP_004533981.1](https://www.ncbi.nlm.nih.gov/protein/XP_004533981.1?report=genbank&log$=prottop&blast_rank=2&RID=NXNVFG1Z015) | 0 | 99 |
|  |  | *D. melanogaster* | AF129079.1 | 0 | 89 |
| *csn3* | 440 | *C.capitata* | [XP_004520211.1](https://www.ncbi.nlm.nih.gov/protein/XP_004520211.1?report=genbank&log$=protalign&blast_rank=7&RID=NXMYZDRW015) | 0 | 91 |
|  |  | *D. melanogaster* | AF129081.1 | 0 | 66 |
| *csn4* | *407* | *C.capitata* | [XP_004522580.1](https://www.ncbi.nlm.nih.gov/protein/XP_004522580.1?report=genbank&log$=prottop&blast_rank=4&RID=NXNMD0CT014) | 0 | 99 |
|  |  | *D. melanogaster* | [NP_477444.1](https://www.ncbi.nlm.nih.gov/protein/NP_477444.1?report=genbank&log$=prottop&blast_rank=22&RID=NXNMD0CT014) | 9e-162 | 84 |
| *csn5* | 333 | *C.capitata* | [XP_004524029.1](https://www.ncbi.nlm.nih.gov/protein/XP_004524029.1?report=genbank&log$=prottop&blast_rank=6&RID=NXNX73HZ014) | 0 | 95 |
|  |  | *D. melanogaster* | [NP_477442.1](https://www.ncbi.nlm.nih.gov/protein/NP_477442.1?report=genbank&log$=prottop&blast_rank=23&RID=NXNX73HZ014) | 0 | 87 |
| *csn6* | 332 | *C.capitata* | [XP_004523378.1](https://www.ncbi.nlm.nih.gov/protein/XP_004523378.1?report=genbank&log$=prottop&blast_rank=5&RID=NXNY1BF6015) | 0 | 91 |
|  |  | *D. melanogaster* | [NP_524451.1](https://www.ncbi.nlm.nih.gov/protein/NP_524451.1?report=genbank&log$=prottop&blast_rank=34&RID=NXNY1BF6015) | 3e-178 | 78 |
| *csn7* | 299 | *C.capitata* | [XP_004534617.1](https://www.ncbi.nlm.nih.gov/protein/XP_004534617.1?report=genbank&log$=prottop&blast_rank=10&RID=NXN29W2X014) | 0 | 84 |
|  |  | *D. melanogaster* | [NP_610379.2](https://www.ncbi.nlm.nih.gov/protein/NP_610379.2?report=genbank&log$=prottop&blast_rank=25&RID=NXN29W2X014) | 5e-118 | 64 |
| *csn8* | 184 | *C.capitata* | XP_004521454.1 | 7e-101 | 78 |
|  |  | *D. melanogaster* | [NP_723378.2](https://www.ncbi.nlm.nih.gov/protein/NP_723378.2?report=genbank&log$=prottop&blast_rank=21&RID=NXS4W1G0014) | 4e-43 | 44 |
| *csn9x1* | 59 | *C.capitata* | XP_012159048.1 | 6e-34 | 88 |
|  |  | *D. melanogaster* | Q7JVR7.2 | 4e-41 | 52 |

*C. capitata: Ceratitis capitata*;

*D .melanogaster: Drosophila melanogaster*
